# 

**Published:** 1973-04

**Authors:** 


					mm
' ' ? -'-mi
Wmm M
?PL.
H
s . i ! ^
3gsfr' - -/-??" &#aBp
;:
JHHH ?? JMI
??11
$
mm
BRISTOL
MEDICO
HIRURGICAL
JOURNAL
asm ~
% n ?"
m
III pip
if? Ajp
=i\ ?
?pi: OF - . |i|':
1$
lil
i'
:?#' :i ?: gPPffigf
***11
1973
VOL. 88 (ii) No. 326

				

